# Quality of technology integration matters: Positive associations with students’ behavioral engagement and digital competencies for learning

**DOI:** 10.1007/s10639-024-13118-8

**Published:** 2024-10-29

**Authors:** Tessa Consoli, Maria-Luisa Schmitz, Chiara Antonietti, Philipp Gonon, Alberto Cattaneo, Dominik Petko

**Affiliations:** 1 Institute of Education, University of Zurich, Kantonsschulstrasse 3, 8001 Zurich, Switzerland; 2 Swiss Federal University for Vocational Education and Training SFUVET, Kirchlindachstrasse 79, 3052 Zollikofen, Switzerland; 3 Swiss Federal University for Vocational Education and Training SFUVET, Via Besso 84 / 86, 6900 Lugano Massagno, Switzerland

**Keywords:** Improving classroom teaching, Information literacy, Secondary education, Evaluation methodologies

## Abstract

Despite extensive research on technology's potential to enhance teaching, large-scale studies often report mixed or negative impacts of technology use at school on student learning achievements. This ambiguity is often attributed to previous large-scale studies focusing more on the frequency rather than the quality of technology integration in the classroom. To further investigate this issue, our study developed the Technology Integration Quality Scale (TIQS) to measure students' perceptions of technology integration across different dimensions of teaching quality: support for learning, classroom management, individualized teaching, and cognitive activation. Using a sample of 2,281 students from 29 upper secondary schools in Switzerland, we validated the TIQS through exploratory and confirmatory factor analyses. We also employed cluster-robust structural equation modelling to examine how both the frequency and perceived quality of technology integration predict students’ self-assessed digital competencies and behavioral engagement for learning. The results show that quality explains considerably more variance than the frequency of technology integration in promoting both students' behavioral engagement and digital competencies for learning. However, for digital competencies, quantity also explains a substantial amount of variance. By simultaneously considering multiple dimensions of teaching quality, the frequency of technology use and two output variables, this study contributes to the existing research by offering a more nuanced perspective on the impact of technology integration. Furthermore, interaction effects between the independent variables highlight the need to further explore the relationships between different dimensions of teaching quality, which could also contribute to the development of the theory of generic teaching quality*.*

## Introduction

While numerous experimental studies and meta-analyses have highlighted the potential of technology to enhance teaching and learning (Chauhan, 2017; Stegmann, 2020; Tamim et al., 2011), large-scale investigations into the impact of technology use in schools on student learning achievements in Switzerland and other countries have yielded mixed and sometimes even negative results (Gerick & Eickelmann, 2014; Konsortium PISA.ch, 2019; Zhu & Li, 2022). In the ongoing debate about the impact of integrating technology into classroom practice, scholars have suggested that the negative findings are due to the fact that these studies have primarily focused on the perceived frequency rather than the perceived quality of technology integration in education (Petko et al., 2017; Juuti et al., 2022; Li & Zhu, 2023). Thus, some researchers have started to focus more on the quality rather than the quantity of technology integration and have proposed different operationalizations of the term (Antonietti et al., 2023; Fütterer et al., 2022; Juuti et al., 2022; Lachner et al., 2024). However, studies analyzing the relationship between the quality of technology integration and students’ learning outcomes often focus merely on the use of technologies to support cognitive activation (Fütterer et al., 2022; Stegmann, 2020). Others have investigated the impact of technology use and teaching quality separately, without using a survey instrument to investigate whether digital technologies are being actively implemented to support teaching quality (J. Wang et al., 2022), and most of these have focused on only one output variable (usually learning achievement or engagement), neglecting other important outcome variables. Moreover, most large-scale studies have not comprehensively investigate the relationships between the frequency of technology use, instructional quality, and multiple outcome variables at all.

The current debate on the quality of technology integration also overlooks crucial aspects that warrant consideration. For instance, an extensive large-scale study (Fraillon et al., 2020) revealed a positive correlation between the frequency of technology use in class and students’ digital literacy in some countries. This finding implies that the frequency of technology use still matters when considering digital literacy as an output variable. Furthermore, the debate often overlooks the fact that PISA studies until 2022 fail to differentiate between the use of technology at school for educational purposes and its use in non-academic activities, such as distraction or entertainment (Konsortium PISA.ch, 2019), which could provide an explanation for the negative results on the impact of the frequency of technology use at school. A study by Bergdahl and Nouri (2020) confirmed that students in technology-enhanced learning settings often use digital technologies to disengage from their educational activities (Bergdahl & Nouri, 2020). In addition, the often single-minded focus on using digital technologies solely to promote cognitive activation is problematic because it overlooks other important dimensions of instructional quality and technology integration. Furthermore, there is a lack of theoretically grounded, reliable, and short survey instruments to assess student perceptions of technology integration across different dimensions of teaching quality (Consoli et al., 2023).

This study was intended to overcome some of the shortcomings of previous research by (1) developing a short survey instrument to measure student perceptions on the role of technology in sustaining different aspects of teaching quality and (2) investigating and comparing how the frequency of technology use *for learning purposes* and the perceived quality of technology integration (i.e., the use of technology to sustain different dimensions of teaching quality) predict students’ self-assessed digital competencies and behavioral engagement for learning. By simultaneously considering multiple dimensions of teaching quality, this study extends understanding of which pedagogical aspects of technology integration are most important for fostering different learning outcomes, thereby informing educational practices and policies regarding pedagogically sound technology integration in schools. Moreover, the development of the Technology Integration Quality Scale (TIQS) based on the Three Basic Dimensions (TBD) framework for general teaching quality (Praetorius et al., 2018) will provide researchers and practitioners with a reliable instrument to assess whether students perceive that teachers’ integration of technology into their teaching practices supports the following dimensions of teaching quality: *support for learning*, *classroom management*, *individualized teaching, and cognitive activation*.

### Defining and measuring technology integration

Technology integration has been conceptualized in different ways, and researchers have developed various instruments and measurement approaches for assessing it. However, in the current discourse, researchers seem to agree that survey instruments assessing technology integration should not solely measure the frequency of technology use but also aspects related to the quality of technology integration in instructional practice (Consoli et al., 2023; Duran, 2022).

A systematic review of survey instruments measuring technology integration in educational research from 2010 to 2021 (Consoli et al., 2023) found that there has been a strong focus on teachers’ rather than students’ views and that ratings on qualitative aspects of technology integration were mostly limited to the cognitive aspects of teaching and learning with technology. Therefore, this study aims to consider different dimensions of teaching quality and analyze the phenomenon from students’ perspectives.

### The three basic dimensions of instructional quality

Teaching quality is an abstract concept with normative and context-related components that is difficult to address theoretically and empirically (Klieme, 2018; Berliner, 2005). Nevertheless, several domain-general dimensions of teaching quality have been identified in previous studies (Creemers & Kyriakides, 2008; Diedrich & Tenorth, 1997; Praetorius et al., 2018). For example, the German TBD framework (Praetorius et al., 2018) recognizes three core aspects of teaching quality that are of pivotal importance across most subjects and contexts: 1) cognitive activation, 2) classroom management and 3) student support. More recently, an extended version of the framework has been developed by considering also subject-specific aspects of teaching quality. This extended version includes the following dimensions: 1) selecting and addressing of contents and subject specific methods; 2) cognitive activation, 3) supporting practicing, 4) formative assessment, 5) learning support for all pupils, 6) socio-emotional support, 7) classroom management (for more details see Praetorius and Gräsel, 2021 and Praetorius, Herrmann, et al., 2020a, 2020b). However, this extended version of the framework is currently still under development, has not yet been empirically tested, and some dimensions appear to be more subject specific than others (Praetorius & Gräsel, 2021). For these reasons, this study focuses on the core dimensions of the well-established and often empirically tested three-dimensional model.

**Fig. 1:**
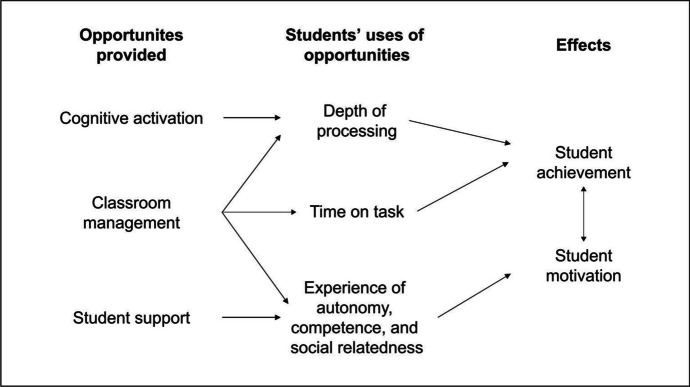
The hypothesized relationships between the TBDs and students’ cognitive and non-cognitive learning outcomes (Praetorius et al., 2020a)

The *cognitive activation* dimension encompasses activities that enhance students’ higher-order thinking skills, foster knowledge construction, encourage metacognitive reflections, and enable students to participate in classroom discussions (e.g., genetic-socratic teaching). The *classroom management* dimension includes aspects such as the absence of disruptions and discipline problems, the structuredness of the instructional practice, and the presence of clear rules and routines. Finally, the dimension of *student support* includes aspects such as the experience of competence, social relatedness, and autonomy (Deci & Ryan, 1985). This last dimension also includes aspects such as differentiation and individualized teaching, which are often associated with the use of technology (Baron et al., 2019). Figure 1 shows how the TBDs are supposed to support psychological factors for learning and how these factors should affect students’ cognitive and non-cognitive learning outcomes.

We decided to develop the TIQS relying on the three dimensional TBD framework because (1) it draws on general theories of education and teaching, and on established research and theories in the field of educational psychology; (2) it is not bound to a specific operationalization or instrument and is therefore adaptable to different contexts—including teaching with digital technologies; (3) it is parsimonious; (4) it is assumed to be applicable in all formal teaching contexts (i.e., across all subjects, grade levels, countries, and cultures); (5) it has been adopted in many large-scale studies (Klieme et al., 2001); (6) several empirical studies using factor analysis have supported the assumption that it is possible to distinguish the three theorized dimensions of teaching quality (Herbert et al., 2022); and (7) and many empirical studies have investigated the impact of the TBDs on different students’ learning outcomes with many positive although still inconclusive results (Praetorius et al., 2018). These findings highlight the necessity for additional research to understand how the TBDs influence or do not influence students’ outcomes.

Several studies have highlighted and explored how digital technologies can support some of the three core dimensions of the TBD framework without referring to the framework. For example, teachers can use digital technologies to support students’ learning by differentiating teaching (Haelermans et al., 2015; Xie et al., 2019), scaffold learners (Jones et al., 2022; Ortel-Cass et al., 2012) or using simulations (Rutten et al., 2012). Teachers can also use technologies to involve students in cognitively engaging learning activities (Stegmann, 2020). By facilitating various teachers’ tasks (Sabanci et al., 2014) and implementing learning management systems (Rubach & Bonanati, 2022), digital technologies can eventually improve classroom management. However, most of these studies have an experimental design or consider only one particular aspect of teaching quality. Few studies consider multiple dimensions of teaching quality simultaneously in the context of the everyday use of digital technologies.

Few researchers have explicitly employed the TBD framework to assess instructional quality in technology-enhanced learning environments (Backfisch et al., 2021; J. Wang et al., 2022; Runge et al., 2023). Quast et al. (2021) developed a four-dimensional scale to assess the extent to which teachers use digital technology to actively promote *cognitive activation*, *structuredness*, *constructive support*, and *differentiation*. However, there is still a lack of research that addresses the perceived quality of technology integration according to the TBD framework from the students' perspective, considering their perceptions and its impact on cognitive and non-cognitive learning outcomes.

Similar to Quast et al. (2021), in order to adapt the TBD framework to a technology-enhanced learning environment, we further considered *individualized teaching* as a dimension of its own, as digital technologies are believed to open up new possibilities to make the learning process more differentiated and personalized (see Lee et al., 2018; Schmid et al., 2022). *Individualized teaching* is a student-centred approach to teaching that seeks to tailor instruction to the experiences, aptitudes, needs and interests of each individual learner (Waxman et al., 2013). Digital technologies, with their range of affordances, are believed to provide several other opportunities for enhancing and transforming teaching and learning practices, such as enabling more immediate and recursive feedback (Cope & Kalantzis, 2017), making learning more collaborative (Iglesias Rodríguez et al., 2017), facilitating self-regulated learning (Bernacki et al., 2011), or providing more opportunities for global citizenship education (Pathak-Shelat, 2017; Truong-White & McLean, 2015). These aspects can also be considered as quality dimensions of technology integration (Consoli et al., 2023), but as they have never been indagated in relation to the TBD framework, and for reasons of parsimony, we have not considered them in our analysis.

### Technology use and teaching quality as factors for students’ behavioral engagement for learning

This study investigates the relationships between the perceived frequency and quality of technology integration and students’ engagement. Following the categorizations of Skinner et al. (2009) and Wong and Liem (2022), we will focus on a specific subdimension of engagement: *students’ behavioral engagement for learning*. This term refers to the intentional exertion of attention and effort toward learning and includes aspects such as diligence, persistence when working on challenging tasks, and actively sustained attention and concentration. Several studies have demonstrated that this subdimension of engagement is frequently associated with desired cognitive learning outcomes, such as learning achievement (Fredricks & McColskey, 2012; Fredricks et al., 2004; Trautwein et al., 2015).

Numerous studies have highlighted the potential positive impact of technology use and technology-enhanced learning environments on students’ engagement or learning achievements (Barana et al., 2020; Schindler et al., 2017; Shi et al., 2020; Tamim et al., 2011; OECD, 2023). Furthermore, several studies have shown that teaching quality is related to students’ engagement and learning achievement (Decristan et al., 2015; Fauth et al., 2014; Hattie, 2008; Leon et al., 2017; Quin et al., 2017; Virtanen et al., 2015). However, few studies have considered technology integration, teaching quality and students’ engagement simultaneously, and thus more research is needed.

One study that examined the relationship between technology use and learning achievement is, for example, the PISA study. The latest PISA results (OECD, 2023) show that, on average, across OECD countries, students who spent up to one hour per day using digital devices for learning activities at school substantially outperformed those who did not use digital devices in mathematics. However, this positive association diminishes when digital devices are used for more than three hours and is no longer observed when devices are used for more than seven hours.

A notable investigation of how different aspects of teaching quality affect learning achievements is Hattie's (2008) synthesis of over 800 meta-analyses. The synthesis found a medium effect (*d* = 0.44) for overall teaching quality, with items related to *cognitive activation* (referred to as “teachers challenging students”) showing the highest correlation (*r* = 0.64), a medium–high effect (*d* = 0.52) for *classroom management*, a high effect (*d* = 0.72) for *student support* (referred to as “teacher-student relationship”), and only a medium–low effect for *individualized instruction* (*d* = 0.23). Recent meta-analyses of technology-supported *individualized teaching* have reached different conclusions. Major et al. (2021) found a small effect (*d* = 0.18), while Zheng et al. (2022) found a heterogeneous medium effect size on learning outcomes and a small heterogeneous effect size on learning perceptions.

Few studies have investigated the relationship between teaching quality and students’ engagement in a technology-enhanced learning environment and came to mixed results. Fütterer et al. (2022) conducted a field study on the relationships between the frequency and quality of technology integration and students’ behavioral engagement in tablet and non-tablet classes. In the subject of mathematics, the study found that the mere frequency of technology use is not significantly related to the development of students’ long-term behavioral engagement, while the experience of high cognitive activation when learning mathematics in tablet classes is. In the subject of German, the study showed a different pattern. Here, the frequency of technology use is significantly related to the development of short- and long-term behavioral engagement, while the experience of high *cognitive activation* is not. J. Wang et al. (2022) investigated the impact of instructional quality (operationalized relying on the TBD framework as *cognitive activation*, *classroom management*, and c*onnectedness*) and the use of technology on students’ engagement. The study found that at the individual level (students), *cognitive activation*, *connectedness*, and the use of technology were significantly and positively associated with students’ engagement and that at the classroom level, students’ shared perceptions of *connectedness* and technology use were positively and significantly associated with their engagement. Furthermore, Fütterer et al. (2022) only focused on the dimension of cognitive activation and both studies did not use a survey instrument to investigate whether digital technologies are used to support teaching quality.

### Technology integration and students’ digital competencies for learning

Technology integration is also commonly considered to foster students’ digital competencies for learning. There are several frameworks and definitions of digital competencies (Ferrari, 2012; e.g. the DigComp 2.2 of Vuorikari et al. (2022) or the Global Framework of Reference on Digital Literacy Skills for SDG indicator 4.4.2 of Law et al., 2018) and different terms used synonymously or closely related to digital competencies (e.g., “digital literacy” Ng (2012); “Computer and Information Literacy” Fraillon et al., 2020). Ng (2012) defines digital literacy within educational contexts as "a broader term that embraces technical, cognitive and social-emotional perspectives of learning with digital technologies, both online and offline" (p. 1066). In the recent literature review on digital literacy in learning and education by Audrin and Audrin (2022) digital learning skills have emerged as a core aspect next to more general digital competencies and 21st-century skills. Students should be able to use digital technologies for a wide range of learning activities. Key aspects are searching, evaluating, and processing information for learning and the ability to use digital technology to collaborate with others.

Multiple studies have emphasized the necessity of teaching digital competencies (for learning) in schools to counter digital inequalities (Hargittai, 2021; Hatlevik et al., 2015; Ren et al., 2022). Contrary to popular belief, so-called digital natives are often unfamiliar with educational technology (Ng, 2012). The International Computer and Information Literacy Study (ICILS) found that roughly one-fifth of students do not reach the first out of four levels of information and computer literacy and that only a few students reach the fourth and highest level (Fraillon et al., 2020). Digital competencies are also expected to play a pivotal role in lifelong learning, which aligns with Goal 4 of the 17 Sustainable Development Goals outlined in the 2030 Agenda (European Commission, 2019b).

Whether and how technology integration in schools actually promotes digital competencies for learning is not entirely clear. The ICILS study showed that in most countries, the frequency of use of ICT applications in class is positively and significantly correlated with higher levels of digital competence(Fraillon et al., 2020). However, the perceived quality of technology integration was not considered in this study.

While many experimental studies have analyzed whether specific educational interventions impact the participants’ information and media literacy (e.g. Weber et al., 2018; Zhang et al., 2022), these studies could not draw conclusions with regard to the general quality of technology integration in everyday classroom settings.

### The present study

Given the lack of an instrument to measure students' perceptions of technology integration across different dimensions of teaching quality (Consoli et al., 2023), the research gap highlighted in the previous chapters, and the need to simultaneously investigate the potential impact of different dimensions of teaching quality on learning outcomes, this study has the following objectives:

1. To develop and examine the factorial validity of the TIQS, which measures students’ perceptions regarding the use of digital technologies to support the following dimensions of teaching quality*: support for learning*, *classroom management, individualized teaching*, and *cognitive activation*.

We expect that the exploratory factor analysis (EFA) will reveal and extract the four underlying factors assumed by our model and that the confirmatory factor analysis (CFA) will validate the factor structure proposed by the EFA. Ideally, CFA will demonstrate a good fit between the observed data and the proposed model.

(2) To investigate and compare how the frequency and perceived quality of technology integration are related to students’ digital competencies and behavioral engagement for learning.

Based on results from previous research, we expect that the frequency of technology use significantly and positively predicts digital competencies for learning (Fraillon et al., 2020) but not behavioral engagement for learning (Fütterer et al., 2022; Juuti et al., 2022) and that the perceived use of technology to support dimensions of teaching quality predicts behavioral engagement for learning.

## Methods

The focus of this study was to construct a short survey instrument to assess teaching quality using technology, as perceived by students. Utilizing data from *N* = 2,281 students across 29 upper secondary schools in Switzerland, we first used EFA and CFA to extract and validate the four-factor structure of the scale. Afterwards, we employed cluster-robust structural equation modeling (Oberski, 2013, 2014) to explore the predictive association between the perceived quality of technology integration and the frequency of technology use (as independent or exogenous variables) and students’ behavioral engagement for learning, and their digital competencies for learning (as endogenous or dependent variables).

### Context

In Switzerland, after attending compulsory schools, about two-thirds of students attend vocational education (which, in most cases, combines a practical apprenticeship within a company and school-based education), and one-third attend a general or specialized baccalaureate school (school-based education only) (Bundesamt für Statistik, 2023). Digital technologies are widely used across all programs. A 2022 survey shows that 89% of upper secondary school students use computers (desktops, laptops, or tablets) at school (SKBF, 2023). Nevertheless, the use of digital technologies for learning purposes does not seem to be widely adopted. PISA 2022 results show that students report using digital technologies at school for learning purposes for less than two hours per day, which is well below the average for OECD countries (OECD, 2023). In most cases, the curriculums of general baccalaureate education and vocational education emphasize the importance of developing students’ digital competencies as a transdisciplinary goal. Some programs also include computer science as a school subject. The use of digital technologies outside of school is also widespread. A national survey (Külling et al., 2022) shows that 99% of young people between the ages of 12 and 19 use cell phones daily or several times a week, 96% use the Internet daily or several times a week, and 91% use social networks daily or several times a week.

### Sample

The data presented in this study comes from a national survey study on digital transformation in upper secondary schools in Switzerland (Antonietti et al., 2023; Petko et al., 2022). The sample used in this study consisted of 2,281 s-year upper secondary school students (age: M = 18.4 years, SD = 4; Md = 17; 52.1% male, 45.1% female, 2.8% other/nonbinary) from 29 schools located in the German-speaking part of Switzerland. The population of interest was all students enrolled in their second Upper secondary school year in the German speaking part of Switzerland (excluding the Canton of Zurich). In total, 55.6% of the participants attended a vocational program, 39% a general baccalaureate program, and 5.4% a specialized baccalaureate school. These percentages indicate a slight underrepresentation of vocational school students in our study compared to the target population.

The data were collected anonymously and voluntarily through an online survey. The survey was conducted using Unipark, a comprehensive online survey platform. The data collection occurred in two phases: the first occurred between September and November 2021 in the canton of Zurich, and the second was carried out between May and August 2022 in the other cantons. There were no pandemic-related restrictions on classroom activities during this period. All upper secondary schools in Switzerland were invited to participate in the study. We sent an e-mail invitation to all school leaders, and if they agreed to participate in the study, we asked them to forward the questionnaire to all second-year students at their schools. About 20% of upper secondary schools participated in the study.

The dataset used in this study consisted of a subset of the whole national dataset (*N* = 8,915) as (1) for accuracy reasons, we wanted to validate the TIQS only in one of the four national languages (German); (2) in the first phase of data collection conducted in the canton of Zurich, we did not consider the dimension of *cognitive activation.* This means that our data can be considered, at best, moderately representative of the German-speaking part of Switzerland (excluding the Canton of Zurich).

During the data cleaning process, we eliminated the data of respondents who filled out the questionnaire too quickly (i.e., two standard deviations above or below average), who did not enter their school code correctly, who were not attending the second year of upper secondary school, and who indicated that none of their teachers used digital technologies in teaching.

### Measures

#### Quality of technology integration

To measure students’ perceptions of teaching quality with digital technologies, we developed the *TIQS,* which includes four dimensions of general teaching quality: *support for learning*, *classroom management*, *cognitive activation,* and *individualized teaching*. To formulate the items, we oriented ourselves to existing operationalizations measuring teaching quality in general (Fauth et al., 2014; Herbert et al., 2022) and teaching quality in the context of technology-enhanced learning (Praetorius et al., 2018; Quast et al., 2021; Wang et al., 2022).

Regarding the *student support* dimension, we focused mainly on cognitive support for learning (like Herbert et al., 2022), which includes actions undertaken by the teacher to facilitate learning and self-efficacy experience among students (Praetorius et al., 2018). Finally, we operationalized the dimensions of *cognitive activation* and *classroom management* more broadly, relying on the operationalizations of Fauth et al. (2014) and Quast et al. (2021). Table 1 shows the English translations of all items.

**Table 1:** Dimensions, subdimensions, and items of the technology integration quality scale (TIQS)

Dimensions	Subdimension	Items
Student’s support	Support for learning	TIQS_SL1 Our teachers use digital media in such a way that difficult topics become more interesting, and we learn more intensively
		TIQS_SL2 Our teachers use digital media in a way that makes it easier for us to understand difficult topics
		TIQS_SL3 Our teachers use digital media in such a way that we can better practice and apply difficult learning goals
Classroom management	Classroom management	TIQS_CM1 Our teachers use digital media in a way that all learners participate in class and there are no disruptions
		TIQS_CM2 When teachers use digital media, learners are less distracted and follow lessons more attentively
		TIQS_CM3 Our teachers use digital media in a way that the lessons are well organized and there are few interruptions
Differentiation	Individualized teaching	TIQS_IT1 Our teachers use digital media in such a way that I receive personal feedback on my learning progress
		TIQS_IT2 Our teachers use digital media in such a way that I get tasks that are not too hard and not too easy for me
		TIQS_IT3 Our teachers use digital media in a way that aligns learning with my own personal interests and learning habits
Cognitive activation	Challenging tasks and higher order thinking skills	TIQS_CA1 When our teachers introduce new topics, they use digital media in a way that makes us reflect deeply
		TIQS_CA2 Our teachers make us use digital media to work on complex tasks for which there is no simple solution
		TIQS_CA3 Our teachers make us use digital media so that we have to actively acquire new knowledge ourselves

The answer options were 1 “The teachers do not use digital technology in the classroom,” 2 “No teachers,” 3 “Few teachers,” 4 “Some teachers,” and 5 “Most teachers.” The first answer option was formulated to identify students who had never been exposed to teachers’ technology use in the classroom. Answer options 2 to 5 were formulated to assess how many teachers, according to students' perceptions, integrate digital technologies in their teaching in a way that supports teaching quality. As this study is only interested in the perceptions of students who are taught using digital technologies, if respondents indicated even once that their teachers do not use digital technologies in the classroom, their responses were excluded from the subset during the data cleaning process. The scale has, therefore, to be considered a four-point Likert scale ranging from 2 to 4. This procedure also allowed us to exclude responses from students who answered inconsistently and, thus, inaccurately.

Since the output variables are not related to a specific subject, and since students in upper secondary school attend different subjects taught by different teachers, we formulated the items and answer options to allow for assessing the overall perceived general quality of teaching with digital technologies across all subjects. While this carries the risk of inaccuracy and over-generalization, it also opens up a new perspective for measuring perceptions of teaching quality and technology integration. In fact, measuring students' overall perceptions of instructional quality or technology integration across all subjects may be particularly valuable for researchers seeking to understand variations in non-subject-specific outcomes, such as digital competencies for learning and students' overall behavioral engagement for learning, which we examined in this study. In addition, this scale—used in conjunction with other scales—could help school leaders or other practitioners assess whether teachers are generally using digital technologies to support the quality of teaching in their schools.

The scale's content validity was assessed through the judgement of experts in the field of technology integration. To assess face validity, feedback was obtained from teachers and students who reviewed the scale.

#### Frequency of technology use

To measure the frequency of technology use for learning purposes during lessons, we calculated the mean of three variables measuring the frequency of computer (desktop, laptop, or notebook), tablet, and smartphone use for learning purposes during lessons (European Commission, 2019a; p. 71). The response format was a five-point Likert scale ranging from 1 (never or almost never) to 5 (several times a day).

#### Students’ self-assessed digital competencies for learning

Students' digital competencies for learning were assessed using an adaptation of Ng’s (2012) self-assessment instrument. We chose this survey instrument because it is short and has shown good reliability and validity properties in previous studies (Ng, 2012; Prior et al., 2016). Ng (2012) conceptualizes digital literacy as a construct that includes technical, cognitive and socio-emotional components. As this study aims to investigate the extent to which students perceive themselves to be able to use digital technologies for learning purposes, we selected (and sometimes reformulated) the items most related to this purpose. Students indicated how much they agree or disagree with the following items: “I know how to use computers effectively for learning” (technical component), “I am able to retrieve information on the Internet and assess whether it is reliable” (cognitive component), “I know how to organize an online group work well” (socioemotional component) and “I know how to get help online if I don't understand something “ (socioemotional component). The answer format was a five-point Likert scale ranging from 1 (strongly disagree) to 5 (strongly agree).

#### Student’s behavioral engagement for learning

Three items previously used in a large-scale survey study in Switzerland (ifes ipes, 2019) were used to measure students’ behavioral engagement for learning. Students indicated how much they agreed or disagreed with the following items: “I try very hard at school,” “When I learn, I do my best,” and “When I study, I continue to work even when the task is difficult.” The answer format was a five-point Likert scale ranging from 1 (strongly disagree) to 5 (strongly agree).

### Data analysis

To validate the TIQS and answer our research questions, we proceeded in four steps. First, to help us select which variables to include in our model and explore the underlying structure of the scales used, we conducted a set of EFA. Second, we tested the factorial validity of the TIQS using confirmatory factor analysis (CFA) (Ziegler & Hagemann, 2015). Third, we calculated the descriptive statistics and reliability estimates of all the scales used in the study. Fourth, to model and test our hypotheses, we used a cluster-robust structural equation modelling approach (Kline, 2023; Oberski, 2013, 2014). As we could not consider the classroom level, this study focuses only on individual-level predictors and students' individual outcomes. The reliability estimates of all scales used in the study were calculated using Cronbach’s alpha (α) and McDonald’s omega (ω) (Hayes & Coutts, 2020).

We conducted the EFA using the *psych* R package (Revelle, 2019). The analyses were performed using maximum likelihood extraction and Promax rotation. We employed Promax rotation, assuming that the factors of the TIQS were correlated. We determined the number of factors using parallel analysis. The confirmatory factor analysis and the structural equation models were investigated using the *lavaan* R package (Rosseel, 2018). Relying on the guidelines of Hu and Bentler (1999), we evaluated the fit of the models using the following goodness-of-fit indices: (1) SRMR (values close to 0.08 or below indicate a good fit), (2) RMSEA (values close to 0.06 or below indicate a good fit), and (3) CFI and TLI (values close to 0.95 or greater indicate a good fit). Chi-square values were reported but not used to assess our models, as the chi-square is notoriously sensitive to the sample size and tends to falsely reject an adequate model in large samples (Brown, 2015; Kyriazos, 2018).

## Results

### Exploratory factor analysis

Based on the results of the EFA, we retained four factors that accounted for 57.8% of the variance in the data. According to our expectations, Factor 1 was characterized by high loadings on items related to *individualized teaching*, Factor 2 by moderate and high loadings on items related to *student support for learning*, Factor 3 by moderate and high loadings on items related to *classroom management*, and Factor 4 by moderate and high loadings on items related to *cognitive activation* (see Table 2). One item (TIQS_CM3) had moderate loadings on both Factors 3 and 4, but we decided to retain it, as, according to our expectations, it had a higher loading on Factor 3. Furthermore, it is highly plausible that several indicators of teaching quality are correlated, as the interplay between the different dimensions is decisive in ensuring teaching quality. We further examined the pattern of factor loadings and found that all items had good or acceptable factor loadings (> 0.4) on their respective factors. Overall, the EFA provided evidence of a four-factor solution that was conceptually meaningful and supported our expectations.

**Table 2:** Factor loadings from EFA

	Factors				
	1	2	3	4	Uniqueness
TIQS_SL1		0.746			0.427
TIQS_SL2		0.976			0.234
TIQS_SL3		0.49			0.428
TIQS_CM1			0.95		0.281
TIQS_CM2			0.563		0.58
TIQS_CM3			0.413	0.341	0.558
TIQS_IT1	0.614				0.503
TIQS_IT2	0.638				0.446
TIQS_IT3	0.959				0.247
TIQS_CA1				0.445	0.475
TIQS_CA2				0.566	0.44
TIQS_CA3				0.828	0.448

### Confirmatory factor analysis

To test the fit of our hypothesized model, we conducted a cluster-robust confirmatory factor analysis. Our model consisted of four latent variables: *support for learning*, *classroom management*, *individualized teaching*, and *cognitive activation*. We used maximum likelihood estimation to estimate the model’s parameters and assessed the model fit using several goodness-of-fit indices. We determined the adequacy of the model fit using the comparative fit index (CFI), Tucker-Lewis index (TLI), root mean square error of approximation (RMSEA), and standardized root mean square residual (SRMR). Given that our data were nested in schools, we used a cluster-robust covariance matrix estimator.

The results of the cluster-robust CFA indicated that our hypothesized model provided a good fit to the data: χ2(48) = 387.755, *p* < 0.001, CFI = 0.97, TLI = 0.96, RMSEA = 0.05, 90% CI [0.05, 0.06], SRMR = 0.03. All the standardized factor loadings were statistically significant and ranged from 0.66 to 0.81 (see Table 3), indicating that the latent variables were highly correlated with their respective indicators.

**Table 3:** Factor loadings from the CFA

Factor	Indicator	Stand. Estimate	SE	*p*
Support for learning	TIQS_SL1	0.761	0.011	< .001
	TIQS_SL2	0.814	0.01	< .001
	TIQS_SL3	0.774	0.011	< .001
Classroom management	TIQS_CM1	0.763	0.012	< .001
	TIQS_CM2	0.655	0.015	< .001
	TIQS_CM3	0.665	0.015	< .001
Individualized teaching	TIQS_IT1	0.723	0.012	< .001
	TIQS_IT2	0.769	0.011	< .001
	TIQS_IT3	0.811	0.01	< .001
Cognitive activation	TIQS_CA1	0.753	0.011	< .001
	TIQS_CA2	0.76	0.011	< .001
	TIQS_CA3	0.674	0.013	< .001

Table 4 displays the correlations between the extracted latent variables. The correlation coefficients are all positive and range from 0.643 to 0.834, which suggests moderate to strong relationships between the four quality dimensions. The strongest correlation was found between *individualized teaching* and *cognitive activation* (r = 0.834; *p* < 0.001), indicating that these two dimensions are more closely related than the other dimensions in the model. This last correlation may raise questions about the discriminant validity of the scale dimensions. However, as high correlations are theoretically expected and fit-values of the CFA are still well above the threshold, it is reasonable to assume a four-factor solution.

**Table 4:** Latent variable correlations

	Support for learning		Classroom management		Individualized teaching		Cognitive activation
Support for learning	1						
Classroom management	0.76	***	1				
Individualized teaching	0.643	***	0.706	***	1		
Cognitive activation	0.768	***	0.789	***	0.834	***	1

^***^ p < *.001*

### Descriptive and reliability analysis

Table 5 shows the internal consistencies, means, standard deviations, intraclass correlation coefficients (ICC) and intercorrelations of all variables considered in the study. All scales showed acceptable or good reliability (McDonald’s ω > 0.7; Cronbach’s α > 0.7). ICCs were calculated using the mean scores of the scales employed in the study and show that school level explains considerable variance in terms of the frequency of technology use for learning purposes at school. The intercorrelation coefficients show a clear pattern: all the dimensions of teaching quality are strongly (r > 0.5), significantly (*p* < 0.001), and positively correlated with each other, whereas all other correlations are significantly (*p* < 0.001), positively, and weakly (r < 0.3) correlated.

**Table 5:** Internal consistencies, means, standard deviations, intraclass correlation coefficients and intercorrelations of all exogenous and endogenous variables

						Correlation matrix						
		ω	α	M (SD)	ICC	1	2	3	4	5	6	7
1	Behavioral engagement	0.803	0.802	3.59 (0.85)	0.0232	--						
2	Digital competencies	0.81	0.801	3.70 (0.85)	0.0383	0.207***	--					
3	Frequency of ICT use	--	--	2.77 (0.77)	0.15	0.098***	0.174***	--				
4	Support for learning	0.83	0.825	3.71(0.71)	0.0184	0.197***	0.2***	0.151***	--			
5	Classroom management	0.74	0.732	3.44(0.72)	0.0266	0.166***	0.18***	0.128***	0.6***	--		
6	Individualized teaching	0.81	0.809	3.09(0.8)	0.0651	0.103***	0.105***	0.114***	0.536***	0.556***	--	
7	Cognitive activation	0.77	0.773	3.41(0.73)	0.0442	0.174***	0.213***	0.158***	0.62***	0.601***	0.659***	--

^*^p < .05, ** *p* < .01, *** *p* < .001

### Structural equation modeling

We investigated the impact of the frequency of technology use and the perceived quality of teaching with digital technologies on students’ behavioral engagement for learning and digital competencies for learning by specifying a structural equation model. We considered the four dimensions of teaching quality and the frequency of technology use as predictors or exogenous variables and students’ behavioral engagement and digital competencies for learning as outcomes or endogenous variables. The tested model showed a good fit: χ2(150) = 683.851,* p* < 0.001, CFI = 0.97, TLI = 0.97, RMSEA = 0.04, 90% CI [0.04, 0.04], SRMR = 0.03. Overall, the model explained 7.3% of the variance in behavioral engagement and 12.4% of digital competencies for learning.

**Fig. 2:**
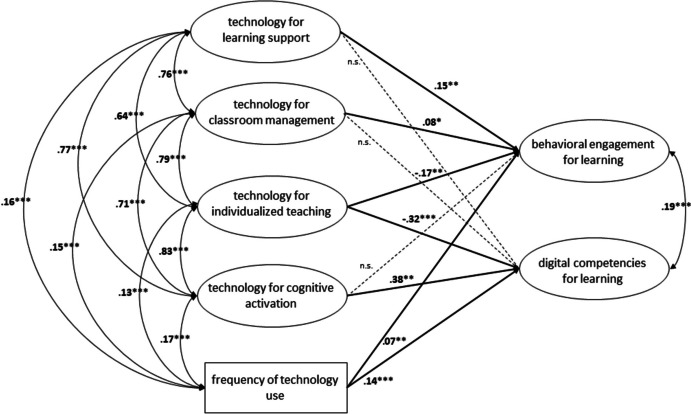
Structural equation model showing the frequency of technology use and the use of technologies to support four different dimensions of teaching quality as predictors for behavioral engagement for learning and digital competencies for learning. * p < .05, ** p < .01, *** p < .001, n.s.: not significant. Standardized estimates

The structural equation model results (see Fig. 2) showed that *student support for learning* (B = 0.18, β = 0.15, SE = 0.07, *p* < 0.01), *classroom management* (B = 0.09, β = 0.08, SE = 0.04, *p* < 0.05), and frequency of technology use (B = 0.07, β = 0.07, SE = 0.03, *p* < 0.01) significantly and positively predicted students’ behavioral engagement for learning. However, *individualized teaching* appeared to have a significant negative impact (B = -0.19, β = -0.17, SE = 0.07, *p* < 0.01). Regarding the prediction of students’ digital competencies for learning, our analysis showed that they were significantly and positively predicted by *cognitive activation* (B = 0.46, β = 0.38, SE = 0.15, *p* < 0.01) and frequency of technology use (B = 0.15, β = 0.14, SE = 0.03, *p* < 0.001). Again, however, *individualized teaching* seemed to have a significant negative impact (B = -0.38, β = -0.32, SE = 0.07, *p* < 0.001).

Since the correlation matrix showed significant and positive correlation coefficients between *individualized teaching* and the two output variables and that the dimensions of teaching quality were highly correlated, we suspected that the negative coefficient of *individualized teaching* in our model could be due to multicollinearity effects (Alin, 2010), mediation effects (MacKinnon et al., 2000), suppression effects (Lancaster, 1999), or other complex interactions between the quality dimensions.

To assess whether our unexpected results were due to multicollinearity effects, we conducted a set of multicollinearity diagnostics (Kim, 2019; Shrestha, 2020). Table 6 shows the initial eigenvalues extracted by principal component analysis with promax rotation. None of the eigenvalues are close to or below 0.05, suggesting the absence of multicollinearity (Shrestha, 2020). Furthermore, all condition indices were less than 10, indicating that multicollinearity is not an issue for these variables (Kim, 2019). We also performed two multiple regression analyses (once for each outcome variable), including all predictors, to assess the VIF and tolerance scores. Interestingly, even in these cases, individualized teaching significantly and negatively predicted the outcome variables. However, all VIF values were well below the threshold of 5 or 10, and all tolerance values were above 0.2, indicating the absence of multicollinearity effects (Kim, 2019).

**Table 6:** Initial eigenvalues and condition indexes from the principal component analysis

Component	Eigenvalue	Condition index	% of Variance	Cumulative %
Support for learning	2.829	1	56.57	56.6
Classroom management	0.96	1.72	19.2	75.8
Individualized teaching	0.487	2.41	9.75	85.5
Cognitive activation	0.403	2.65	8.06	93.6
Frequency of use	0.321	2.97	6.42	100

To further investigate the reasons for these unexpected negative coefficients, we examined the relationship between each quality dimension and the outcome variables separately and tested a model with ‘overall technology integration quality’ as a second-order latent factor. In this way, we aimed to (1) obtain more information about the relationship between each quality dimension and the outcome variables, without the influence of other quality dimensions, (2) detect potential suppression or mediation effects by highlighting changes in the direction or strength of the relationships when the dimensions are considered in isolation, compared to when they are part of a more complex model, and (3) examine how all four quality dimensions together predict the outcome variables. All these could help interpret our findings and guide further theory development and hypothesis refinement. However, while examining each quality dimension separately could provide valuable insights, it is essential to remember that the ultimate goal is to understand how these variables operate together. The results of those analyses must, therefore, be interpreted with caution.

**Fig. 3:**
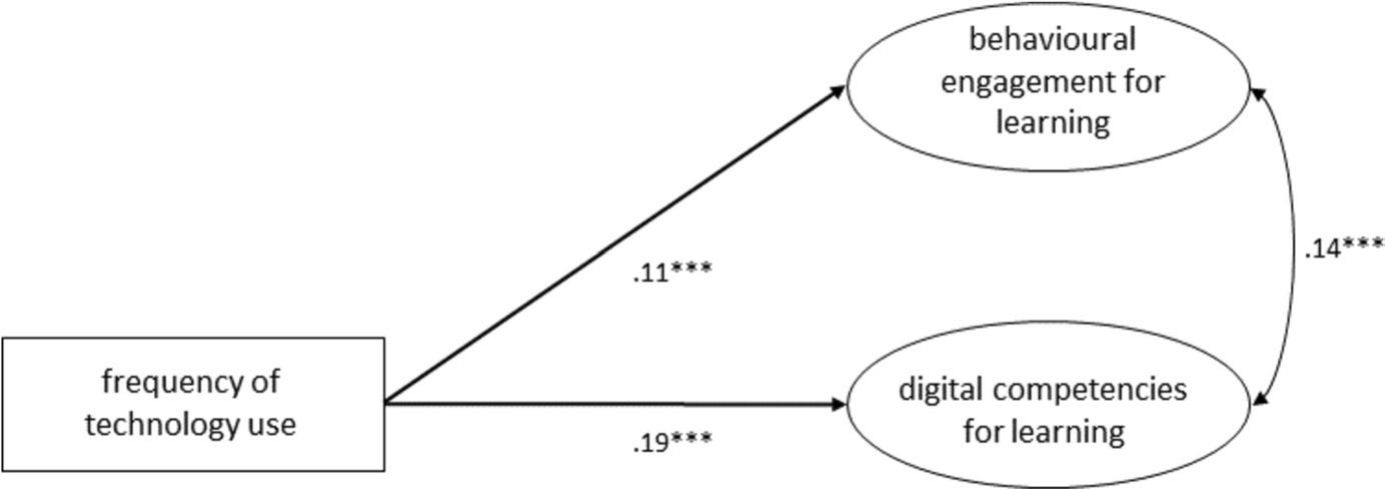
Structural equation model showing the frequency of technology use as predictor for behavioral engagement for learning and self-assessed digital competencies for learning. * p < .05, ** p < .01, *** p < .001. Standardized estimates

Since, for the purposes of this study, it is crucial to distinguish between the variance explained by the frequency of technology use alone, and that explained by the perceived quality of technology integration, we always included the frequency of technology use in the models. We started by specifying a model with only the frequency of technology use as a predictor (see Fig. 3), which served as a baseline. This model fitted the data well (χ2(18) = 122.429, p < 0.001, CFI = 0.98, TLI = 0.97, RMSEA = 0.05, 90% CI [0.05, 0.06], SRMR = 0.03) and explained 1.2% of the variance in behavioral engagement and 3.7% of digital competencies for learning.

**Fig. 4:**
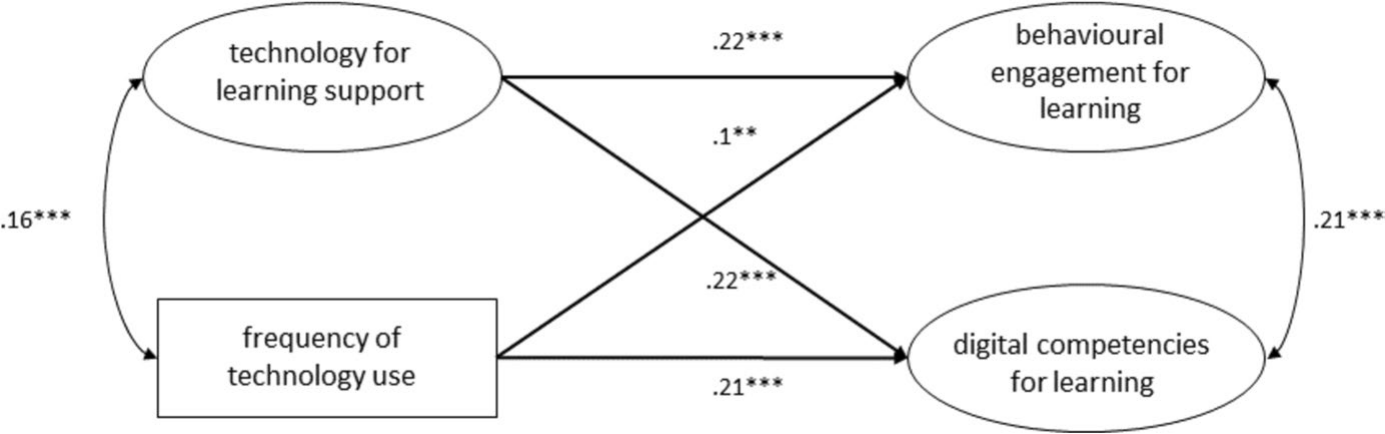
Structural equation model showing the frequency of technology use and the use of technologies to support students’ learning experience as predictors for behavioral engagement for learning and self-assessed digital competencies for learning. * p < .05, ** p < .01, *** p < .001. Standardized estimates

The structural equation model used to investigate the dimension of *cognitive support* (see Fig. 4) fitted the data well (χ2(39) = 150.445, p = 0.000, CFI = 0.99, TLI = 0.98, RMSEA = 0.03, 90% CI [0.03, 0.04], SRMR = 0.03) and showed that both students’ *support for learning* (B = 0.26, β = 0.22, SE = 0.04, *p* < 0.001) and frequency of technology use (B = 0.08, β = 0.1, SE = 0.03, p < 0.01) significantly and positively predicted students’ behavioral engagement for learning. Also students’ self-assessed digital competencies for learning were significantly and positively predicted by *cognitive support for learning* (B = 0.26, β = 0.22, SE = 0.04, *p* < 0.001) and frequency of technology use (B = 0.16, β = 0.21, SE = 0.02, *p* < 0.001). This model explained 6% of the variance in behavioral engagement and 8.2% of self-assessed digital competencies for learning.

**Fig. 5:**
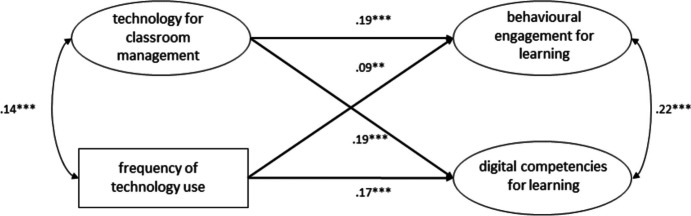
Structural equation model showing the frequency of technology use and the use of technologies to support classroom management as predictors of behavioral engagement for learning and self-assessed digital competencies for learning. * p < .05, ** p < .01, *** p < .001. Standardized estimates

The model we utilized to investigate the dimension of *classroom management* (see Fig. 5) showed a good fit (χ2(39) = 194.171, *p* < 0.001, CFI = 0.98, TLI = 0.97, RMSEA = 0.04, 90% CI [0.03, 0.04], SRMR = 0.03) and showed that both *classroom management* (B = 0.20, β = 0.19, SE = 0.03, *p* < 0.001) and frequency of technology use (B = 0.08, β = 0.09, SE = 0.03, p < 0.01) significantly and positively predicted students’ behavioral engagement for learning. Students’ digital competencies for learning were also significantly and positively predicted by both *classroom management* (B = 0.20, β = 0.19, SE = 0.03, *p* < 0.001) and frequency of technology use (B = 0.17, β = 0.17, SE = 0.02, *p* < 0.001). This model l explained 4.9% of the variance in behavioral engagement and 7.2% of self- assessed digital competencies for learning.

**Fig. 6:**
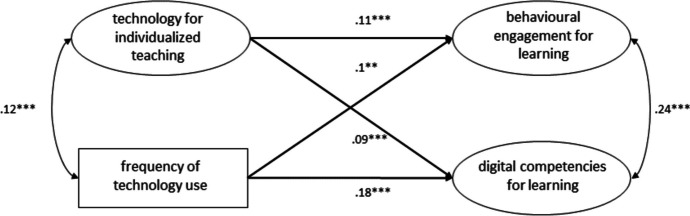
Structural equation model showing the frequency of technology use and the use of technologies to support individualized teaching as predictors for behavioral engagement for learning and self-assessed digital competencies for learning. * p < .05, ** p < .01, *** p < .001. Standardized estimates

The structural equation model employed to investigate the dimension of *individualized teaching* (see Fig. 6) fitted the data well (χ2(39) = 180.391, p = 0.000, CFI = 0.98, TLI = 0.98, RMSEA = 0.04, 90% CI [0.03, 0.04], SRMR = 0.03) and showed that both *individualized teaching* (B = 0.13, β = 0.11, SE = 0.03, *p* < 0.001) and frequency of technology use (B = 0.1, β = 0.1, SE = 0.02, p < 0.001) significantly and positively predicted students’ behavioral engagement for learning. *Individualized teaching* (B = 0.11, β = 0.09, SE = 0.03, *p* < 0.01) and frequency of technology use (B = 0.19, β = 0.18, SE = 0.03, *p* < 0.001) also significantly and positively predicted students’ self-assessed digital competencies for learning. This model explained 2.5% of the variance in behavioral engagement and 4.5% of self-assessed digital competencies for learning.

**Fig. 7:**
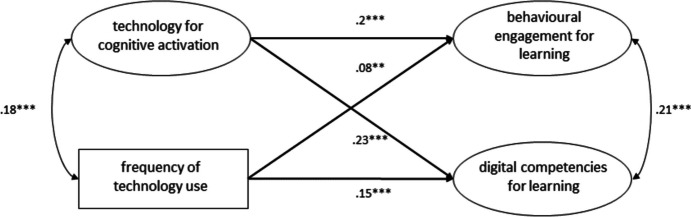
Structural equation model showing the frequency of technology use and the use of technologies to support students’ cognitive activation as predictors for behavioral engagement for learning and self-assessed digital competencies for learning. * p < .05, ** p < .01, *** p < .001. Standardized estimates

The model we utilized to investigate the dimension of *cognitive activation* (see Fig. 7) also showed a good fit (χ2(39) = 197.162, p = 0.000, CFI = 0.98, TLI = 0.97, RMSEA = 0.04, 90% CI [0.04, 0.05], SRMR = 0.03). Both *cognitive activation* (B = 0.24, β = 0.2, SE = 0.03, *p* < 0.001) and frequency of technology use (B = 0.08, β = 0.08, SE = 0.02, *p* < 0.01) significantly and positively predicted students’ behavioral engagement for learning. *Cognitive activation* (B = 0.29, β = 0.23, SE = 0.06, *p* < 0.001) and frequency of technology use (B = 0.16, β = 0.15, SE = 0.03, *p* < 0.001) also significantly and positively predicted students’ self-assessed digital competencies for learning. This model explained 5.1% of the variance in behavioral engagement and 9% of self-assessed digital competencies for learning.

**Fig. 8:**
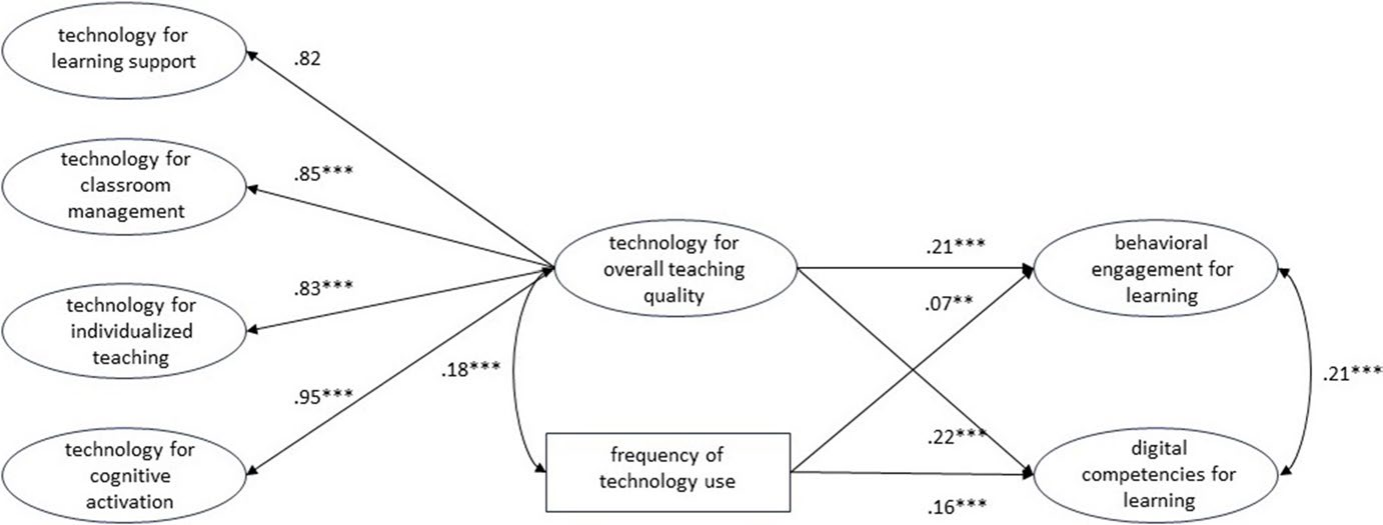
Structural equation model showing the frequency of technology use and the overall technology integration quality as predictors for behavioral engagement for learning and self-assessed digital competencies for learning. * p < .05, ** p < .01, *** p < .001. Standardized estimates

Finally, to assess how the four quality dimensions collectively predict the two output variables, we tested a model with a second-order latent factor (see Fig. 8). The model fitted the data well (χ2(161) = 716.144, p = 0.000, CFI = 0.97, TLI = 0.96, RMSEA = 0.04, 90% CI [0.04, 0.04], SRMR = 0.03). The overall quality of technology integration (B = 0.31, β = 0.21, SE = 0.04, p < 0.001) and frequency of technology use (B = 0.07, β = 0.07, SE = 0.03, p < 0.01) both significantly and positively predicted students' behavioral engagement in learning. The overall quality of technology integration (B = 0.33, β = 0.22, SE = 0.06, p < 0.001) and frequency of technology use (B = 0.16, β = 0.16, SE = 0.03, p < 0.001) also significantly and positively predicted students' self-rated digital competencies for learning All the standardized factor loadings were statistically significant and ranged from 0.82 to 0.95 (see Fig. 8), indicating that the second-order latent factor was highly correlated with the first order factors. The model explained 5.6% of the variance in behavioral engagement and 8.4% of the variance in self-rated digital competencies for learning.

## Discussion

This study was designed to accomplish two primary objectives. The first was the development and validation of the TIQS. The second was to analyze (1) the relationship between student perceptions of technology integration across different dimensions of teaching quality (support for learning, classroom management, individualized teaching, and cognitive activation), (2) the frequency of technology use for learning at school, and (3) two crucial outcome variables: students’ behavioral engagement for learning and students’ self-assessed digital competencies for learning.

The TIQS validation process involved EFA and cluster-robust CFA. EFA showed evidence for a four-factor solution that supported our expectations, and our hypothesized CFA model fit the data well. These findings suggest that the TIQS is a valid instrument for measuring four dimensions of technology integration quality and confirm the multidimensionality of teaching quality hypothesized by the TBD framework, even in a technology-enhanced learning context (see also Quast et al., 2021; Wang et al., 2022).

To analyze the relationship between the four dimensions of technology integration quality and the outcome variables, a cluster-robust structural equation modeling approach was employed. This approach allows for a comprehensive examination of multiple variables within a single model by considering the clustered structure of the data (students within schools). The results of the SEM analyses provided valuable insights into the relationship between technology integration practices and students’ behavioral engagement and self-assessed digital competencies for learning.

Supporting our expectations, our findings indicated that the use of technology to support students in their learning experiences (support for learning) is a crucial factor influencing behavioral engagement for learning. The use of digital technologies to better manage and structure lessons (classroom management) was also found to predict this outcome variable significantly and positively. Contrary to our expectations, we found that the frequency of technology use also significantly and positively predicted students’ behavioral engagement for learning (although the variance explained was almost negligible). In addition, cognitive activation showed no significant relationship, and individualized teaching was found to be a significant negative factor.

Regarding students’ self-assessed digital competencies for learning (and according to our expectations), the findings highlighted the importance of technology use to support cognitive activation. Additionally, the frequency of technology use for learning was found to be a significant positive factor, although the variance explained by this predictor was considerably lower than that explained by the quality dimensions. Unexpectedly, classroom management and support for learning showed no significant relationship with the output variable, and, as in the case of the prediction of behavioral engagement for learning, individualized teaching also significantly and negatively impacted self-assessed digital competencies for learning.

The different dimensions of teaching quality were examined separately, and each showed a positive and significant relationship with the outcome variables. This implies that the nonsignificant and negative relationships observed in the model with all four quality dimensions are due to interactions between the dimensions (i.e., moderation or suppression effects). These findings highlight the need to further explore the complex interactions between the different dimensions of technology integration quality and teaching quality. These interaction effects could also help explain the inconsistent results often seen in teaching quality research (Praetorius et al., 2018). Future research on technology integration should move away from overly simplistic approaches focusing solely on measuring a single quality dimension (e.g., cognitive activation) and seek to model the interactions between different dimensions of technology integration quality and teaching quality (see also Alp Christ et al., 2024).

In particular, the individualized teaching dimension was found to be subject to interaction effects, being a positive and significant factor when analyzed in isolation and a negative and significant factor when analyzed simultaneously with the other quality dimensions. As Table 4 shows, individualized teaching is particularly highly correlated with cognitive activation; we believe that the negative effect could be due to a mediating effect of cognitive activation ‘absorbing’ part of the explained variance of individualized teaching. This seems to be particularly plausible for the prediction of the self-assessed digital competencies for learning, where the coefficient for cognitive activation is exceptionally high and the coefficient for individualized teaching is negative. Theoretically, this could be explained by the fact that differentiation (or individualized teaching) is an indispensable condition for cognitive activation. Thus, the variance explained by well-implemented individualized teaching (which supports students’ cognitive activation) is entirely ‘absorbed’ by cognitive activation. On the other hand, the variance explained by poorly implemented individualized teaching, which does not lead to cognitive activation, shows its negative effect on the output variables. Indeed, in the MAIN-TEACH model of teaching quality hypothesized by Charalambous and Praetorius (2020), the differentiation dimension was conceptualized as “the grounding layer of all dimensions, acknowledging the importance of differentiation and adaptation for all teacher actions and interactions with students” (p. 5). This means that this dimension must be present, at least in part, to ensure teaching quality in all the other dimensions. The MAIN-TEACH model (Charalambous & Praetorius, 2020) could also explain why classroom management and learning support did not significantly affect the output variables, as they were conceptualized as conditions for cognitive activation and other quality dimensions. However, the MAIN-TECH model does not seem to explain the prediction of behavioral engagement for learning. In this case, it seems more plausible that the dimension support for learning acted as a mediator, absorbing the variance of the other dimensions. This hypothesis is supported by studies showing that the support dimension typically predicts affective learning outcomes (e.g., student interest in mathematics; see Klieme et al., 2001). Future studies should further explore these interactions to better understand the relationship between the use of technology to support different dimensions of teaching quality and student learning outcomes.

In general, compared to the variance explained by the model with only the frequency of technology use as a predictor (shown in Fig. 3), all structural equation models examined in this study that included one or more dimensions of teaching quality explained at least twice the variance of behavioral engagement for learning. Except for two models (shown in Figs. 5 and 6), they also explained twice the variance of self-assessed digital competencies for learning. Furthermore, all structural equation models that included one or more dimensions of teaching quality were able to explain considerably more variance in the output variables. These results indicate that student perceptions of the integration of technology across different dimensions of teaching quality are indeed more influential than the frequency of technology use for learning. The results of our study also highlight that the frequency of technology use for learning, if considered alone, predicts a negligible variance of behavioral engagement for learning (the model displayed in Fig. 3 predicts 1.2% of the variance). By contrast, the frequency of technology use seems to predict a considerable amount of variance (3.7%) of self-assessed digital competence for learning. These results are very important for practitioners and contribute to current debate on the quantity and quality of technology integration (Petko et al., 2017; Fütterer et al., 2022; Juuti et al., 2022). Several previous studies have shown that the frequency of technology use correlates with digital self-efficacy, which in turn correlates with digital competencies (Fraillon et al., 2020; Rohatgi et al., 2016). In contrast to the study by Fraillon et al. (2014), which reported a positive but not significant relationship between the frequency of technology use at school and students’ computer and information literacy (see also Gerick et al., 2017), our study found that the frequency of technology use for learning purposes at school can be significantly and positively correlated with digital competencies for learning. This may be due to our different operationalizations of the variables considered in this study, to issues related to self-assessment (e.g., overestimation of own competencies), to characteristics of the Swiss context, or to the fact that the pedagogical/educational quality of technology integration has improved in recent years.

In general, the impact of using digital technologies to support the four dimensions of teaching quality measured by the TIQS does not appear to explain a great amount of variance in the output variables. There are several possible explanations for this. First, the TIQS measures the perceived quality of technology integration across all subjects, with students asked to provide an average assessment considering all their teachers and school subjects. As a result, we obtained moderate values that tended to gravitate toward the middle. Despite this, our analyses still successfully identified four factors and provided the first evidence for the predictive validity of different quality dimensions, suggesting that this approach remains valid. Second, the TIQS focuses on only four dimensions of teaching quality using digital technologies. It is plausible that additional dimensions not captured by our scale could further explain the variance in the outcome variables. Lastly, our statistical models were not designed to account for all the variance observed in the outcome variables. Our aim was to determine whether the perceived use of digital technologies to support the dimensions of teaching quality has a statistically significant impact on these variables. We recognize that other variables, such as overall teaching quality or student motivation, may exert even greater influences on the outcome variables.

A strength of this study lies in the dataset collected within an authentic context. Notably, certain discrepancies between the results obtained in our study and those from other studies (often employing experiments, field studies, or surveys with small sample sizes) may be attributed to this aspect. The inclusion of a larger and more diverse dataset in our study allowed for a deeper understanding of the phenomenon under investigation.

## Limitations

This study has a number of limitations that should be taken into account when interpreting the results. First, the study design employed a cross-sectional approach, which limited our ability to establish causality or the temporal sequence of events. Longitudinal studies could provide a more robust understanding of the relationships among the investigated variables over time (Alp Christ et al., 2024).

Second, our analysis could not consider the classroom level. The students’ data used in this study were nested in classes and schools. Through a cluster-robust analysis, we were able to consider the school level. Unfortunately, due to practical constraints related to the different organizations of the schools, we could not consider the classroom level, which plays a relevant role in research on teaching quality (Fauth et al., 2014; Köhler et al., 2020). Future research should aim to consider the classroom level to capture the impact of classroom-level factors on the observed outcomes.

Another limitation arises from the voluntary participation of the participants, which could have introduced self-selection bias. However, given that our sample was quite large and represented both vocational and general baccalaureate schools, we believe that the results obtained can still be reasonably generalizable, at least for the German-speaking region of Switzerland.

Another limitation is that to avoid excessive complexity in the study, we did not consider some critical control variables, such as students’ gender, socioeconomic background, and learning performance. Studies have shown that males tend to overestimate their digital competencies (Hargittai & Shafer, 2006), and having a higher socioeconomic status positively affects digital competencies (Hatlevik et al., 2018; Scherer & Siddiq, 2019). Furthermore, there is evidence suggesting that learning performance and related constructs, such as scientific literacy, are positively correlated with or predicted by digital competencies (Hatlevik et al., 2015; Hübner et al., 2023; Lei et al., 2021) and students’ learning engagement (Fredricks & McColskey, 2012; Fredricks et al., 2004; Trautwein et al., 2015). Students’ learning engagement also seems to be related to variables such as motivation, confidence in their own capacities, and perceptions of the school environment (Deci et al., 1991; M.-T. Wang & Eccles, 2013). By including these control variables, future research could gain a more nuanced analysis and better identification of the underlying mechanisms at play. However, considering the complex interplay of these factors in multilevel structural equation modeling would require an even larger dataset.

Additionally, reliance on students’ self-reported data introduces potential biases and limitations. Self-report measures may be subject to bias, such as social desirability, which can affect the accuracy and reliability of the data. Combining self-report data with objective measures or triangulating data from multiple sources would strengthen the validity of the results. Moreover, the items of the TIQS were formulated in an abstract manner (we asked the students to evaluate the overarching quality of technology integration). This abstract formulation may overlook some context-specific nuances (e.g., related to a specific school subject). Future studies could try to adapt the scale to a specific context.

We would also like to emphasize that we have only focused on four specific aspects related to the quality of technology integration (*students’ support for learning*, *classroom management*, *individualized teaching*, and *cognitive activation*) and have not considered essential aspects, such as ethical issues, the exploitation of the affordances of new technologies, or the fostering of media education (for an overview on aspects related to the quality of technology integration, see Consoli et al., 2023).

Future studies could also contribute to further investigate the validity and reliability of the scale, for example, by investigating the scale’s predictive, convergent and discriminant validity, by testing scalar invariance in different groups of students, or by assessing the reliability of the TIQS at the school level (Aditomo & Köhler, 2020).

## Conclusion and implications

From a practical perspective, the results of this study show that the perceived quality of technology integration is more influential than the frequency of technology use in promoting two learning outcomes: behavioral engagement for learning and self-assessed digital competencies for learning. However, in the case of self-assessed digital competencies for learning, the frequency of technology use also explains considerable variance. These findings underline the importance of focusing on the quality of technology integration, without forgetting the importance of the frequency of technology use for learning purposes in developing learners’' digital competencies.

Beyond those practical considerations, this study contributes to the ongoing development of the theory of generic teaching quality (Praetorius et al., 2020a). First, this study highlights the importance of modelling and theorizing the relationships between different dimensions of teaching quality. Second, it provides empirical support for the structural hypothesis that multiple dimensions of teaching quality can be distinguished in different contexts, such as the technology-enhanced learning environment studied here. Finally, our study reveals inconsistencies in the impact of quality dimensions on student outcomes, which the structure of the relationship between the quality dimensions could perhaps explain. A meta-analysis of the impact of the TBDs on student outcomes (Praetorius et al., 2018) also reported inconsistent findings. As highlighted by Praetorius et al. (2020a, 2020b), these findings warrant further discussion and should inform potential revisions to the theory of teaching quality.

Furthermore, this study significantly advances understanding of the complex relationship between technology integration and student learning outcomes. It contributes to the current body of empirical research on the impact of the quality and quantity of technology integration on student learning outcomes (Fütterer et al., 2022; Juuti et al., 2022; J. Wang et al., 2022) by providing a more nuanced and accurate perspective on the evaluation of technology integration in the classroom. Indeed, the simultaneous inclusion in the analysis of different dimensions of technology integration quality, along with the frequency of technology use for learning purposes at school (in contrast to the general frequency of technology use at school) and the consideration of two output variables, provides a broad and differentiated understanding of the complex relationships involved. In addition, compared to other instruments developed to assess the quality of technology integration, the TIQS allows for a more accurate assessment of whether a teacher’s technology integration in classroom practices actively and directly supports the quality of teaching as perceived by learners.

In addition to advancing knowledge, our study developed and validated the TIQS to assess student perceptions of technology integration across different dimensions of general teaching quality. This instrument can be considered a valid and reliable tool that can be used to further investigate these issues in technology-enhanced learning environments.

## Data Availability

Data will be made available in a data repository in December 2024.

## Declarations

This work was founded by the Swiss National Science Foundation, related to project number 187277.
